# A Comparison of EIS and QCM NanoMIP-Based Sensors for Morphine

**DOI:** 10.3390/nano11123360

**Published:** 2021-12-11

**Authors:** Roberta D’Aurelio, Ibtisam E. Tothill, Maria Salbini, Francesca Calò, Elisabetta Mazzotta, Cosimino Malitesta, Iva Chianella

**Affiliations:** 1 Surface Engineering and Precision Centre, School of Aerospace, Transport and Manufacturing, Cranfield University, Cranfield, Bedford MK43 0AL, UK; i.tothill@cranfield.ac.uk (I.E.T.); maria.salbini@nanotec.cnr.it (M.S.); francesca.calo@unisalento.it (F.C.); 2 Laboratorio di Chimica Analitica, Edificio Multipiano CSEEM A6., Dipartimento di Scienze e Tecnologie Biologiche ed Ambientali, Università del Salento, I-73100 Lecce, Italy; elisabetta.mazzotta@unisalento.it (E.M.); cosimino.malitesta@unisalento.it (C.M.)

**Keywords:** electrochemical impedance spectroscopy (EIS), interdigitated electrode (IDE), screen printed electrode (SPE), quartz crystal microbalance (QCM), molecularly imprinted polymer (MIP), morphine

## Abstract

In this work we have compared two different sensing platforms for the detection of morphine as an example of a low molecular weight target analyte. For this, molecularly imprinted polymer nanoparticles (NanoMIP), synthesized with an affinity towards morphine, were attached to an electrochemical impedance spectroscopy (EIS) and a quartz crystal microbalance (QCM) sensor. Assay design, sensors fabrication, analyte sensitivity and specificity were performed using similar methods. The results showed that the EIS sensor achieved a limit of detection (LOD) of 0.11 ng·mL^−1^, which is three orders of magnitude lower than the 0.19 µg·mL^−1^ achieved using the QCM sensor. Both the EIS and the QCM sensors were found to be able to specifically detect morphine in a direct assay format. However, the QCM method required conjugation of gold nanoparticles (AuNPs) to the small analyte (morphine) to amplify the signal and achieve a LOD in the µg·mL^−1^ range. Conversely, the EIS sensor method was labor-intensive and required extensive data handling and processing, resulting in longer analysis times (~30–40 min). In addition, whereas the QCM enables visualization of the binding events between the target molecule and the sensor in real-time, the EIS method does not allow such a feature and measurements are taken post-binding. The work also highlighted the advantages of using QCM as an automated, rapid and multiplex sensor compared to the much simpler EIS platform used in this work, though, the QCM method will require sample preparation, especially when a sensitive (ng·mL^−1^) detection of a small analyte is needed.

## 1. Introduction

The development of sensors for the detection of small molecules is a challenge for researchers. Most small molecules, such as drugs, toxins and environmental pollutants, require an affinity-based platform for their detection. Therefore, selecting the appropriate transducer/instrumentation, sensing layer and assay format is vital for the sensitivity and selectivity required for the targeted application. Sensors are analytical devices developed to detect a single or multiple molecules and can be utilized in several diagnostic settings, such as medicine, food, security and environmental science. The main components are: (1) a molecular receptor (also known as the sensing receptor), able to bind or recognize the analyte of interest; (2) a transducer, able to convert the binding event into a signal; (3) a processor, able to handle the signal such that it can be visualized and analyzed through a dedicated software.

Sensing receptors play a central role in fabricating the sensor surface, resembling molecular receptors present in the living systems. When successfully developed (artificial or semi-artificial receptor) or selected (natural receptor) sensing receptors can bind the target analyte with a degree of sensitivity and specificity and can prompt the sensor response. Examples of biological or bio-inspired receptors are peptides [1,2], antibodies [3,4], whole cells [5], bacteriophages [6,7], DNA [8,9], nanobodies [10,11], affibodies [12,13], aptamers [14], molecularly imprinted polymer nanoparticles (nanoMIP), MIP layer [15,16], and aptamer-MIP hybrid receptors [17,18]. Briefly, a MIP is a polymer with binding sites complementary in shape, size and functional groups to the target analyte (template). The synthesis approach can be classified as covalent, non-covalent or semi-covalent according to the type of binding occurring between the template and the functional monomer(s). The most common MIP synthesis methods are precipitation polymerization, emulsion polymerization and bulk polymerization [19]. In contrast, electropolymerization is widely used to produce MIP films [20]. MIP can be synthesized at micro/macroscale or nanoscale, such as nanoparticles (nanoMIP), nanofilms and bulk molecularly imprinted polymers (MIPs).

MIP-based sensors are considered a viable strategy to overcome the fragility of sensors based on antibodies and other natural based receptors (such as aptamers) and can be applied in harsh environmental conditions (e.g., temperature fluctuation, an environment with denaturing agents) [15]. Many types of transducers can be used to develop sensor platforms, such as electrochemical (amperometry, potentiometry, conductimetry and impedimetric) [16,21], mass sensitive (piezoelectric or acoustic wave, mechanical) [22], or optical (colorimetric, fluorescence, luminescence, interferometry, spectroscopy of optical waveguides and surface plasmon resonance) [23]. Generally, the choice of the most appropriate transducer is dictated by the final application (e.g., for lab-based or in-field use), as well as the molecular weight of the target analyte (small molecule, protein, whole-cell) and the range of the detection limits (bulk or trace analysis). Likewise, the selected transducer may influence the assay design (e.g., direct, indirect, competitive). The potential for miniaturization, ease of use, robustness and portability of the transducer become critical when the sensor platform has to operate in a wide variety of environments. Under these circumstances, bulky surface plasmon resonance (SPR) systems (e.g., Biacore, Biolin Scientific, Gamry) are less favored than miniaturized and portable instruments (e.g., PalmSense). The sensor sensitivity, specificity, and selectivity can be further enhanced by engineering the sensor surface and developing *ad-hoc* sample preparation and assay method. Specifically, surface functionalization and blocking combined with the assay design play a role in achieving adequate sensor selectivity and sensitivity. With this respect, the advent of nanomaterials and their application in the surface functionalization and assay design offers an effective strategy in achieving outstanding sensor performance. 

EIS as a sensing platform has seen an increase in popularity due to the many advantages over other biosensors, i.e., miniaturization feasibility, low production cost, on-site application opportunity and the low limit of detection (in the order of nM/pM). EIS biosensors measure the impedance at the electrode/solution interface and take into account both types of currents: capacitive (C) and resistance (R) [24]. The change in the impedance components (charge transfer resistance, Rct and double layer capacitance, Cdl) directly expresses the solution composition or the binding occurring at the electrode/solution interface. Specifically, the Rct value can change due to the variations of the charges distribution onto the sensor surface and other factors, such as the change in the hydrophobicity of the surface. EIS can be performed with or without an electrochemical reaction occurring in the solution, thus resulting in the faradaic and non-faradaic processes, respectively. The Faradaic EIS sensor was used in this work as it results in a sensitive electrochemical sensor able to detect changes in resistance charge transfer (Rct) due to the binding events between nanoMIP and morphine. Usually, [Fe (CN)_6_]^3−/4−^ ions are used as a negatively charged redox probe, while hexaammineruthenium III/II ions are used as positively charged redox probes. The binding events occurring at the electrode surface can be monitored by the change in Rct. Indeed, the binding events result in a thicker sensing layer, thus hindering the contact between the electrolyte solution and the electrode surface [25]. As it can be inferred, the larger/more concentrated the analyte, the thicker the sensor layer becomes, which, consequently, increases the Rct value. Furthermore, the analyte or the sensing layer charges may influence the Rct signal since it can induce repulsive or attractive forces according to the charge of the ions in the probing solution. Overall, the Rct is the parameter more often measured. In recent work [16], we have demonstrated the use of the Faradaic EIS sensor with nanoMIP as the sensing receptor for the detection of cocaine.

On the other hand, piezoelectric sensors have been widely explored in combination with MIPs. The quartz crystal microbalance (QCM), the sensor platform used in this work, is classified as a bulk acoustic wave (BAW). The QCM sensor, also named acoustic resonator, resonates at a particular frequency (ƒ, Hz), which is sensitive to the mass changes at the sensor surface [26]. When the resonator is immersed in a liquid and the viscoelastic materials (such as nanoMIP) are attached to the sensor surface, the frequency change is proportional to the fluid’s viscosity and the density. The phenomenon is described by Kanazawa and Gordon equation–derived from the Sauerbrey equation, which defines the relation between the frequency and the mass changes when measurements are carried out in the gas phase [27]. In the presence of a liquid, the oscillation frequency is dissipated by the damping effect occurring at the sensor surface boundaries and induced by the soft layer’s viscosity and density. When the buffer composition is kept constant throughout the measurements, the viscosity of the liquid (buffer solution) is considered constant. In contrast, the analyte’s concentration dissolved in the buffer solution changes the density and the viscosity at the sensor surface, thus yielding the frequency changes of the QCM s response. In other words, the binding signal arises from a difference in resonant frequency due to the permanent sensor interface’s viscosity and density changes, i.e., the receptor-analyte binding.

EIS and QCM sensing platforms are often used to characterize and develop sensors for detecting small molecules [28,29]. The two platforms have also been developed as a point of testing devices, e.g., QCM-I Mini (Gamry Instruments, Warminster, PA, USA), BluQCM Q (BioLogic, Seyssinet-Pariset, France), Palmsense4 (PalmSens BV, Houten, The Netherlands), AnaPot EIS (Zimmer & Peacock AS, Horten, Norway). The EIS is mainly used to assess the functionalization or degradation of the sensor surface, whereas the MIP based EIS sensor has been used to detect different molecular sizes, including small molecules. To the best of our knowledge, a direct comparison of QCM and EIS nanoMIP sensors in detecting small molecules as morphine has never been reported. This work presents a comparison of EIS and QCM sensors functionalized with the same nanoMIP to detect morphine (MW = 375.84 g·mol^−1^)–although two slightly different assay designs were used. The two sensors were compared concerning surface functionalization, assay design, sensitivity and specificity. The results provide the opportunity to discuss the advantages and disadvantages of applying nanoMIP EIS and QCM platforms for detecting small molecules such as drugs.

## 2. Materials and Methods

### 2.1. Reagents

Morphine and cocaine nanoMIP in acetonitrile were provided by Prof Piletsky’s group (University of Leicester, Leicester, UK) [30]. During the synthesis, nanoMIP were functionalized with primary amino groups, thus allowing their covalent attachment onto the sensor surface. Cocaine hydrochloride, morphine hydrochloride (trihydrate) and levamisole were purchased from Merck KGaA (Darmstadt, Germany) and were handled according to the Home Office (London, UK) guidelines. 3-(N-Morpholino)propanesulfonic acid powder (MOPS) was purchased from Sigma Aldrich and used to make buffer solutions. 11-Mercaptodecanoic acid (11-MUDA) was also purchased from Merck KGaA and was dissolved in 50 mL of ethanol (pure ethyl alcohol, anhydrous, ≥99.5%) at a concentration of 5 mM. N-Hydroxysuccinimide (NHS) and 1-ethyl-3-(3-dimethylaminopropyl) carbodiimide (EDC) were purchased from Thermo Fisher Scientific (Rugby, UK) and dissolved in water to obtain 0.1 M and 0.4 M solutions, respectively. Ethanolamine (MEA, 1 M), bovine serum albumin (BSA, 10%, *w/v*) and Tween 20 (1%, *v/v*) were purchased from Merck KGaA and used as blocking agents. Tris buffer, sodium chloride (NaCl), and 40 nm gold colloidal (AuNPs, ~7210 particles mL^−1^, OD = 1) were purchased from Merck KGaA. The 10 mM redox couple solution ([Fe(CN)6]^3−/4−^) was prepared by dissolving potassium ferrocyanide (K_4_[Fe(CN)_6_]) and potassium ferricyanide (K_3_[Fe(CN)_6_]) in MOPS (10 mM, pH 7.4).

All the reagents were of analytical grade. All the aqueous solutions were prepared using ultrapure water (18 MΩ-cm) and were filtered through a Whatman^®^ nitrocellulose filter 0.2 µm (Whatman International Ltd., Maidstone, UK) or a 0.2 µm Corning^®^ syringe filter (Corning Inc., New York, NY, USA) before use. All the buffers prepared for QCM experiments were degassed before use. NanoMIP solutions were filtered with a 0.45 µm Corning^®^ syringe filter (Corning Inc., New York, NY, USA). All apparatus and measurements settings are reported in the Supplementary Materials (Section S1).

### 2.2. EIS Sensor Assembly

NanoMIP EIS sensors were fabricated using identical functionalization protocol on both DropSens screen-printed electrodes (SPE) and interdigitated electrodes (IDE) (both from Metrohm, Runcorn, UK). Briefly, the gold electrodes were first cleaned according to the manufacturer instructions and then incubated overnight in an ethanol solution containing 5 mM 11-MUDA to create a self-assembly monolayer (SAM). Afterwards, the sensors were rinsed with deionized water and dried with nitrogen. A mixture of EDC/NHS was used to activate the carboxylic groups of 11-MUDA, thus enabling the attachment of the nanoMIP (2.4 mg·mL^−1^) via amine coupling chemistry [16]. Morphine nanoMIP were left in contact with the activated surface for 1 h at 23 °C (±1). Then, the electrodes were again rinsed with water and dried under a gentle nitrogen stream. Any remaining activated carboxylic groups were capped with 1 M ethanolamine dissolved in MOPS (10 mM) and adjusted at pH 8.5. The rest of the sensor surfaces was blocked by a mixture of 0.1% (*w/v*) of BSA and 1% (*v/v*) of Tween 20.

### 2.3. EIS Sensor Cumulative Assays

#### 2.3.1. EIS-SPE Sensor

Cumulative concentrations assays were performed using morphine concentrations, in the range of 100 pg·mL^−1^–50 ng·mL^−1^, prepared in three different buffers, 10 mM MOPS pH 6.0 and pH 7.4 and PBS pH 9.0. The buffer used to prepare the washing buffer (1.2 mL), the diluent for the blank sample (50 µL) and the redox couple solution (50 µL, 10 mM [Fe (CN6)]^3−/4−^) were adjusted accordingly. Control sensors were investigated to confirm that the analyte was binding to the morphine nanoMIP. For this, cumulative assays were also carried out on sensors functionalized without the nanoMIP (control sensor) using the optimized buffer and pH conditions. 

For each experiment, a 20 µL of each concentration was left in contact with the sensor surface for 30 min. The electrode surface was then rinsed with 1.2 mL of washing buffer and dried under a gentle stream of N_2_. The EIS reading was thus performed and recorded in the presence of the redox couple solution ([Fe(CN)_6_]^3−/4−^). To assess the sensors specificity, the sensor response towards morphine was compared to the sensor response towards paracetamol and cocaine with assays performed in the optimized conditions. All the experiments were carried out on independent sensors and at least in triplicates.

#### 2.3.2. EIS-IDE Sensor

The cumulative assay on EIS-IDE sensor was performed following the same protocol optimized using the SPEs with minor differences. Morphine was dissolved in MOPS (10 mM, pH 7.4) and diluted in the concentration range of 10 pg·mL^−1^–50 ng·mL^−1^. EIS measurements were performed using 10 µL of redox couple solution (10 mM [Fe (CN_6_)]^3−/4−^) dissolved in MOPS buffer (10 mM, pH 7.4). The electrode surface was washed with 600 µL of washing buffer and dried under a gentle stream of N_2._ Incubation time and EIS reading were as for the optimized assay performed on SPEs.

#### 2.3.3. EIS Data Analysis

The experimental data were fitted onto an appropriate equivalent circuit by EIS spectrum analyser^®^ v1.0, and the obtained Rct (Ω) value was expressed as a percentage of the blank signal (expressed as −Δ% Rct, when negative values were obtained), thus standardizing the sensor response across the sensors [10]. When cumulative assays were performed, the obtained Δ% Rct values were plotted against the analyte concentrations. A linear regression curve was achieved by expressing the concentration on a LOG_10_ scale. The slope of the calibration curve, the coefficient of determination (R^2^) of the linear regression, and the significance (*p*-value ≤ 0.05) was considered to assess the sensors performances.

### 2.4. QCM Sensor Assembly

The surface of the gold QCM chip was cleaned carefully with piranha solution. Briefly, the QCM chips were covered with 2–3 mL of freshly prepared piranha solution (3:1 H_2_SO_4_:H_2_O_2_). After 15 min, the piranha solution was carefully discarded and the surface of the sensor was washed with a large amount of double-filtered deionized water until neutralization was reached. The QCM chips were then rinsed with ethanol and dried under a gentle stream of nitrogen. The cleaned QCM chips were incubated overnight in the dark into a solution of 11-MUDA (50 mL, 50 mM) dissolved in pure ethanol. Subsequently, the chips were rinsed with ethanol, dried with nitrogen and docked into the QCMA-1 instrument. The functionalization of the QCM sensor surface was performed using PBS (pH 7.4) as a running buffer (degassed and filtered), while the flow rate was 25 µL min^−1^ for the whole procedure. A protocol previously developed in our laboratory was used to perform the nanoMIP functionalization [3,31,32]. Briefly, the MUDA carboxylic groups were activated by injecting 100 µL of a freshly mixed EDC/NHS solution (0.4 M EDC and 0.1 M NHS dissolved in water) for 4 min. Then, 100 µL of morphine nanoMIP (1.2 mg·mL^−1^) suspended in PBS (pH 7.4) was injected for another 4 min onto spot 1, thus allowing the attachment via amine coupling. Soon after, 100 µL of cocaine nanoMIP (1.8 mg·mL^−1^) was injected onto spot 2, thus obtaining the nanoMIP QCM sensor. The non-reacted carboxyl groups were capped by injecting 75 µL of ethanolamine (1 M, pH 8.5) in PBS for 3 min. 

### 2.5. QCM Sensors Cumulative Assay

The morphine AuNPs cumulative assay was performed on multiplex nanoMIP QCM sensor, with morphine nanoMIP immobilized onto spot 1 and cocaine nanoMIP immobilized onto spot 2. The temperature was set at 25 °C and the flow rate was kept as 25 µL·min^−1^ throughout the assay duration. The assay was performed using PBS (pH = 7.4) as the running buffer. The nanoMIP QCM sensor chip was docked into the QCM instrument and was primed several times with the running buffer until a stable sensor baseline was reached.

Cocaine hydrochloride trihydrate and morphine hydrochloride trihydrate were conjugated to 40 nm gold nanoparticles (AuNPs), as described in Section S4 of the Supplementary Materials [3,33]. Bare AuNPs, blank AuNPs (no drugs, but blocked with BSA), cocaine AuNPs and morphine AuNPs were all suspended in doubled distilled water to assess the success of the conjugation by dynamic light scattering (DLS). All measurements were performed having gold nanoparticles as reference materials and doubled distilled water as eluent. At least ten measurements were recorded per each sample. The data was collected and statistically analyzed to reveal differences in the hydrodynamic diameter, *d*_H_ (nm), across the investigated samples. To do this, one-way ANOVA test with Sheffe’s post hoc test was applied.

**Figure 1:**
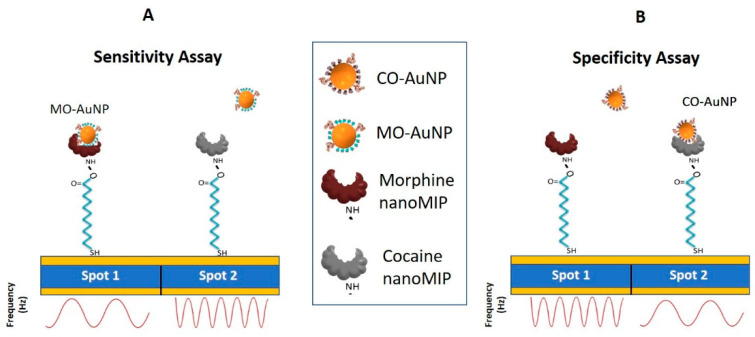
Schematic of sensitivity(**A**) and specificity(**B**) assays performed using multiplex nanoMIP QCM sensor.

Morphine AuNPs, cocaine AuNPs, and the blank AuNPs were suspended in PBS (pH 7.4) to perform the cumulative assays. For sensitivity assay, serial dilutions of morphine AuNPs (250 ng·mL^−1^–50 µg·mL^−1^) were prepared in PBS (pH 7.4) as diluent. At least three injections of the blank solution were performed until a stable blank signal was achieved. The blank solution was composed of blank AuNPs suspended in PBS (pH 7.4). Then, 100 µL of increasing concentrations of morphine AuNPs were injected onto both spots 1 and 2 for four minutes at the flow rate of 25 µL min^−1^. The QCM sensor response, in terms of frequency (Hz), was recorded in a time-resolved mode. Similarly, the specificity assay was carried out by injecting increasing concentrations of cocaine AuNPs, while the working conditions were kept the same as with the morphine assay. A schematic of sensitivity and specificity assays are presented in Figure 1.

Experiments were conducted in triplicates, and the data were collected and analyzed using the dedicated software (Sierra Analyser v.3.1.10.0, Sierra Sensors GmbH, Hamburg, Germany). Firstly, the signals from control and blank tests expressed in frequency (Hz) were subtracted from the sensor response signal. Then, the frequency value recorded at the end of each injection was subtracted from the frequency value recorded before the injection, thus achieving the actual change of the sensor response, ΔF (Hz). The ΔF was then expressed as % of the highest ΔF (Hz) value obtained during the assay:

(1) $$\%\;\Delta F=\frac{\Delta F_{x}}{\Delta F_{max}}\times 100$$

The average (SD±) of the % ΔF values were then plotted against the increasing concentration of morphine, thus obtaining the standardized non-linear calibration curve, which was transformed in a linear regression curve by expressing the drug concentrations in LOG_10_.

### 2.6. Statistical Analysis and Limit of Detection Equation

Statistical analyses of the results were carried out using Excel^®^ (Microsoft^®^, Microsoft Corporation, Redmond, WA, USA) and SPSS^®^ Statistics 24.0 (IBM^®^, Armonk, NY, USA) software. The significance level (*p*-value) was set at 0.05. Parametric or non-parametric statistics were applied as appropriate. Specifically, the one-way ANOVA test and post-hoc analysis were applied to assess differences between two or more datasets. 

The following Equation (2) was applied to calculate the limit of detection (LOD):

LOD = [(3 × SD Blanks) − b]/m,(2)

where b, is the y-intercept of the linear calibration curve; m, is the slope of the linear calibration curve.

## 3. Results and Discussion

### 3.1. Morphine NanoMIP Characterization

Several batches of nanoMIP, produced using the solid-phase approach, were supplied by the University of Leicester (Prof. Piletsky’s group). Once received, the nanoMIP batches were characterized by DLS and transmission electron microscopy (TEM) (details are listed in Section S2 in the Supplementary Materials). The DLS analysis (Table S1A) indicated that the average hydrodynamic diameter (*d*_H_) across four batches was equal to 171.75 ± 66.65 nm. The polydispersity index (PDI) values were below 0.30 for all the DLS readings, thus confirming the purity and narrow distribution of the nanoMIP samples. The one-way ANOVA and post-hoc Scheffé’s test (Table S1B) revealed a minimal variation of the *d*_H_ within the batches, likely ascribed to human error during the synthesis. Specifically, the *d*_H_ of batch 1 and batch 4 were not significantly different. On the other hand, there was a difference between batch 1 and batches 2 and 3. Nevertheless, the particle’s concentration and solvation and the ionic strength of the dispersant may affect the estimation of the *d*_H_ due to the agglomeration/aggregation phenomena and corona formation around the solid nanoparticle. For these reasons, the resulting *d*_H_ value is often higher compared to the real one [34]. Therefore, Batch 2 nanoMIP size and shape were investigated using TEM analysis. As shown in Figure S1, nanoMIP appears as round particles with diameters ranging from 213.75 nm to 295.08 nm, and an average size value (SD±) equal to 250.16 nm (±24.30 nm). The TEM results and SD± were consistent with the *d*_H_ value of DLS result of batch 2 (268.39 ± 37.38 nm). Cocaine nanoMIP characterization study has been already reported in our previous publication [16].

### 3.2. EIS nanoMIP Sensors

#### 3.2.1. Assembly and Surface Characterization Study

The morphine nanoMIP sensor was fabricated onto SPEs and IDEs. Morphine nanoMIP, at a concentration of 2.4 mg·mL^−1^ suspended in a mixture of acetonitrile/water (*v/v*, 3:1), were covalently immobilized onto the gold electrodes by amine coupling. As EIS is used to assess modifications occurring at the surface boundaries, each sensor functionalization step (i.e., from cleaned bare electrodes up to surface blocking) was probed using this technique. In addition, the 3D printed CACIDE cable holder was designed and manufactured in-house to stabilize the IDE sensor connection (Section S3, Figures S2 and S3) and to allow performing reliable EIS measurements. 

**Figure 2:**
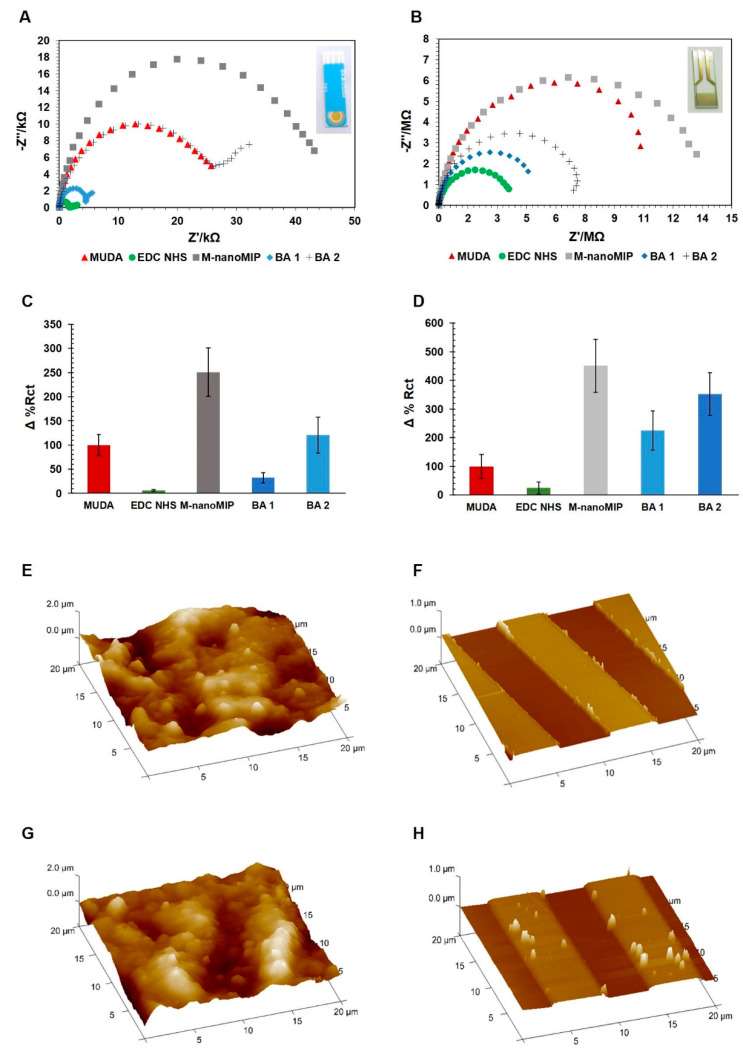
Nyquist plots obtained during morphine nanoMIP EIS sensor fabrication using DropSens SPE. (inset) (**A**) and DropSense IDE (**B**): the electrode coated with MUDA (red), EDC/ENS activation (green), nanoMIP attachment (grey), ethanolamine blocking (blue). Morphine nanoMIP = 2.4 mg·mL^−1^; BA 1 = Ethanolamine pH 8.5; BA 2 = 0.1% BSA − 1% Tween 20. Average of Δ% Rct values (SD±) obtained at each sensor fabrication point performing EIS analysis onto DropSense SPE and IDE (**C** and **D**, respectively). Error bars refer to the SD± (nm) of replicates (*n* = 6). AFM 3D images of the DropSens SPE and IDE surface topography before (**E**,**F**) and after (**G**,**H**) the morphine nanoMIP deposition.

The EIS functionalization spectra of both SPE and IDE were reproducible and consistent with previous results [16], as shown in Figure 2A–D. The attachment of the nanoMIP onto the SPE and IDE sensor surface was also investigated by AFM. The 3D topography images before and after nanoMIP functionalization were compared for both SPE (Figure 2E,G) and IDE (Figure 2F,H) electrodes. Overall, the AFM confirmed the success of nanoMIP attachment onto both electrodes’ surface, although the surface was not fully saturated. This is desirable as a fully saturated surface will create an insulating layer, suppressing the EIS signal. In addition, the 3D images of IDE electrodes revealed a generally smother gold surface.

#### 3.2.2. NanoMIP EIS-SPE Sensor 

Morphine, the analyte selected for this study, is a small molecule (mw 375.84 g·mol^−^^1^) and acts as a zwitterion, having two hydroxyl groups and one tertiary amide group. The pH can affect the ionization of these functional groups, thus changing the overall charges distribution of morphine as the pH approaches the pka (6.13) or the pkb (9.85) (values from PubChem, pubchem.ncbi.nlm.nih.gov/compound/Morphine accessed on 5 December 2021). The pH value also plays a role in the binding between the nanoMIP and the analyte. Therefore, the morphine cumulative assay was carried out using MOPS or PBS (10 mM) at a neutral pH value (MOPS, pH 7.4), a slightly acidic pH (MOPS, pH 6.0) and a basic pH (PBS, pH 9.0) values, thus to assess the effect of the pH on the sensitivity of the morphine nanoMIP sensor. The test at pH 9.0 was carried out using PBS, as this pH value is outside the MOPS buffer working range. Subsequently, the EIS data were fitted into a Randles equivalent circuit, and the Rct values were extrapolated. The average Rct error (%) and the χ^2^ demonstrating the quality of the fitting are reported in Table 1. The Rct values were expressed as −Δ% Rct and were used to plot the non-linear and the linear calibration curve.

**Figure 3:**
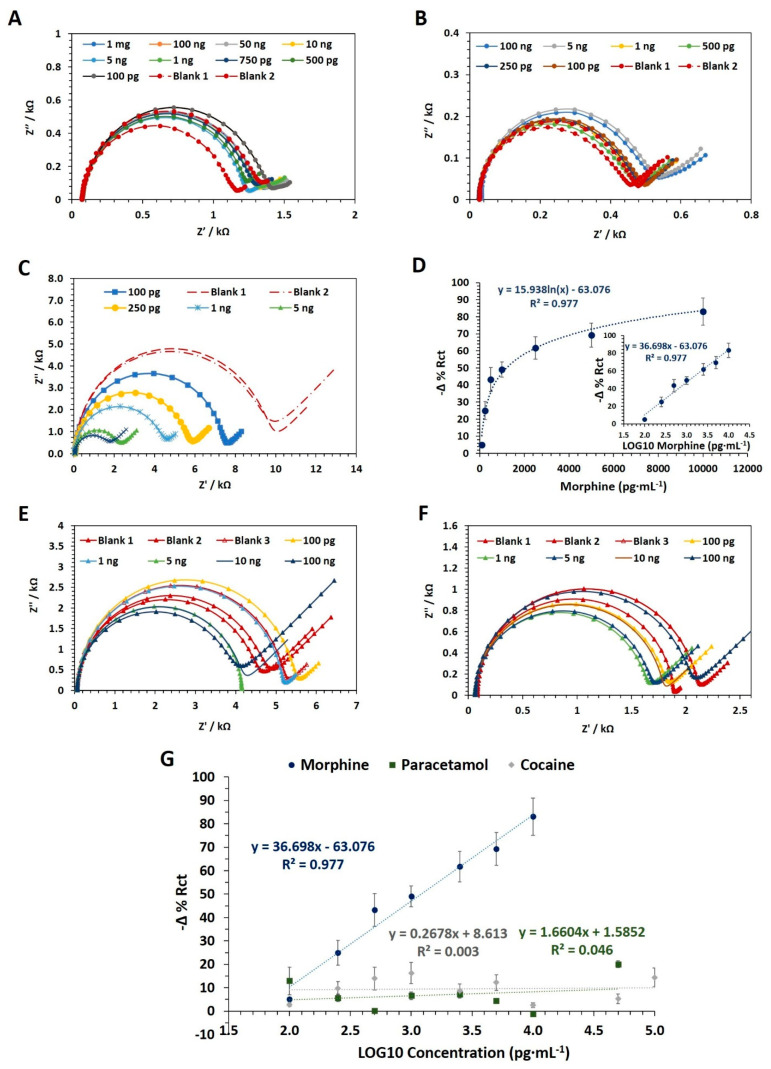
Nyquist plots of the data obtained during the morphine cumulative assay performed at pH 6.0 (**A**), 9.0 (**B**), and 7.4 (**C**). (**D**) The non-linear and linear (inset graph) calibration curves related to the morphine cumulative assay performed at pH 7.4 (100 pg·mL^−1^–50 ng·mL^−1^) performed onto several independent morphine nanoMIP EIS sensors (fabricated using SPE). The morphine nanoMIP at the concentration was equal to 2.4 mg·mL^−1^. Error bars refer to the standard deviation of replicates (*n* = 6). Nyquist plots of the data obtained during the paracetamol (**E**) and cocaine (**F**) specificity assay performed at pH 7.4 onto several independent morphine nanoMIP EIS sensors (fabricated using SPE). (**G**) Comparison between cocaine, paracetamol and morphine linear calibration curves and corresponding R^2^ values.

The EIS data of the cumulative assays performed on SPE indicated that the Rct (Ω) did not vary at increasing concentrations of morphine dissolved in MOPS (10 mM) and in PBS (10 mM) at pH 6.0 and 9.0, respectively (Figure 3A,B). On the other hand, when the cumulative assay was performed at pH 7.4, a decrease in Rct values (Ω) as morphine concentration increased was observed (Figure 3C). Notably, the statistical analysis revealed a positive correlation between −Δ% Rct and increasing analyte concentrations (r = 0.986, *n* = 7, *p*-value < 0.0005). Furthermore, as reported in Figure 3D, the R^2^ of the calibration curves was equal to 0.977 (*p*-value < 0.0005), whereas the LOD was calculated as 0.11 ng·mL^−1^. The morphine cumulative assay at pH 7.4 was replicated using control sensors (i.e., the sensor fabricated without the morphine nanoMIP) to prove that the morphine was binding to the nanoMIP. The sensor response fluctuated between 5 and 25 −Δ% Rct and was not correlated to the increasing morphine concentration (r = 0.600; *n* = 5; *p*-value = 0.250), confirming that morphine binds to the nanoMIP immobilized on the sensor surface. Therefore, MOPS at pH 7.4 was considered optimal and was applied in further investigations.

The sensor specificity was evaluated versus paracetamol, one of the most common morphine cutting agents and cocaine, another illicit drug commonly abused. For the experiments, cocaine and paracetamol were suspended in MOPS (pH 7.4), and concentrations in the range of 100 pg·mL^−1^–100 ng·mL^−1^ were prepared. The results indicated that the sensor response (−Δ% Rct) did not significantly increase while increasing the concentration of paracetamol and cocaine (Figure 3E,F, respectively). The statistics are reported in Table 1. In addition, the morphine nanoMIP EIS sensor response against paracetamol, cocaine, and morphine were plotted together and displayed in Figure 3G to highlight the difference. 

#### 3.2.3. EIS-IDE NanoMIP Sensor

**Figure 4:**
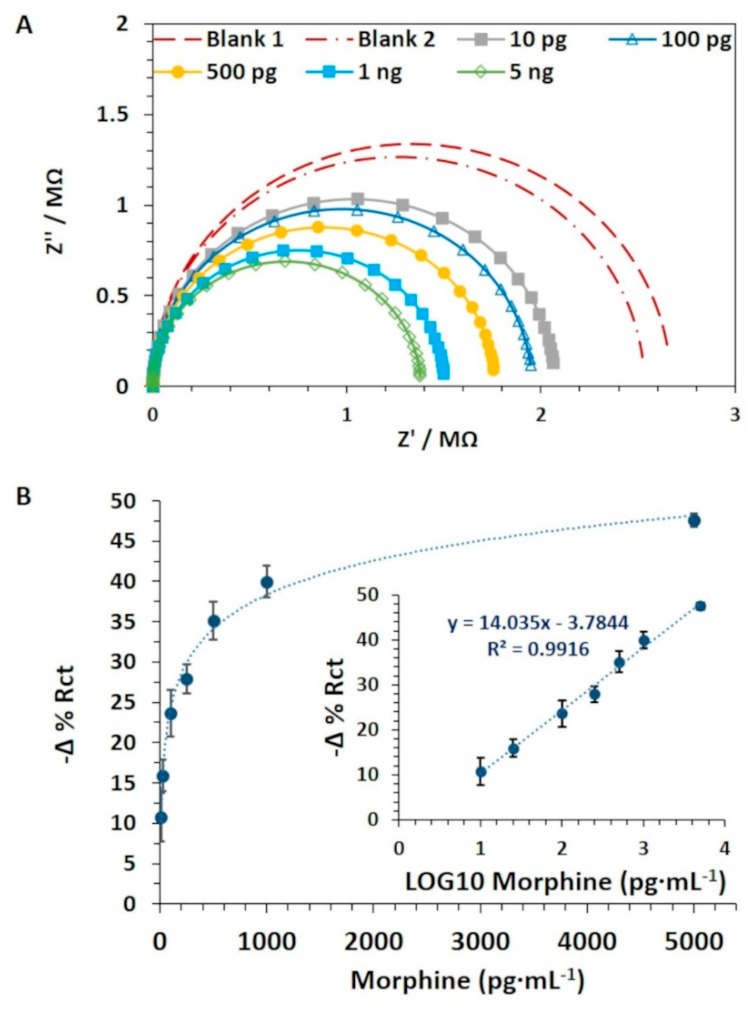
(**A**) Nyquist plots data obtained during the morphine cumulative assay performed at pH 7.4 (100 pg·mL^−1^–50 ng·mL^−1^) using morphine nanoMIP EIS sensors (fabricated on IDE). (**B**) The non-linear and linear (insert graph) calibration curves related to the morphine cumulative assay performed onto the morphine nanoMIP EIS sensor (fabricated on DropSens IDE). The sensors were fabricated using morphine nanoMIP at a concentration equal to 2.4 mg·mL^−1^. Error bars refer to the standard deviation of replicates (*n* = 4).

Interdigitated electrodes are known to possess a higher sensitivity than standard SPE electrodes [35]. Therefore, the sensitivity of the morphine sensor fabricated on IDE was evaluated by performing the morphine cumulative assay. Similarly to previous experiments, sensors were fabricated onto IDE electrodes using morphine nanoMIP at a concentration of 2.4 mg·mL^−1^. The concentration of the nanoMIP was kept constant to allow a direct comparison with the SPE sensor. However, because of the higher sensitivity of IDE electrodes, the morphine concentrations (dissolved in 10 mM MOPS, pH 7.4) ranged from 10 pg·mL^−1^ to 50 ng·mL^−1^. The resulting EIS data (Figure 4) were fitted using the simplified Randles equivalent circuit.

The achieved χ^2^ and %Rct Error values (SD±) were equal to 0.0052 (±0.0013) and 2.03% (±0.46), respectively, thus confirming a good fit of the selected model with the measured EIS data. The −Δ% Rct values were obtained and were plotted against the morphine concentrations, expressed as pg·mL^−1^. The results showed a positive correlation (r = 0.996; *n* = 7; *p*-value < 0.0005) between the sensor response (−Δ% Rct) and the morphine concentrations in the linear range of 10 pg·mL^−1^–5 ng·mL^−1^. Signal saturation was observed at a concentration above 5 ng·mL^−1^. The non-linear and linear calibration curves were plotted, and the R^2^ of the calibration curve was equal to 0.992 (*p*-value < 0.0001). The LOD was as low as 0.11 ng·mL^−1^. Therefore, both the morphine nanoMIP sensors developed on the IDE and the SPE achieved the same LOD and were able to detect morphine at trace levels. The selectivity study was not replicated on IDE as a similar result to that obtained on SPE was expected since the same nanoMIP and chemistry was used to immobilize the receptor and block the sensor surface. In addition the IDE used for the work were expensive and brittle (the electrode substrate is thin glass), which often resulted in electrodes breakage, making the repetition of the selectivity study on the IDE electrodes financially unappealing. 

### 3.3. QCM NanoMIP Sensor

#### 3.3.1. QCM Sensors Assembly

The QCM sensing platform used in this work has an integrated and automated microfluidic system, which can be operated with minimal human intervention. The system allows programmable injections onto the docked nanoMIP-QCM sensor surface, thus permitting real-time response to different samples injected during the cumulative assays. All the cumulative assays were performed on freshly fabricated nanoMIP-QCM sensor chips and in a direct assay format. The cumulative assay was first performed using analyte dilutions only (i.e., drugs were not conjugated to AuNPs). However, as no sensor response was detected in the ng-µg·mL^−1^ range, the drugs were conjugated to gold nanoparticles (AuNPs) to increase the drugs’ molecular mass and enhance the sensor signal [3]. The success of the AuNP conjugation was investigated by DLS analysis and results are presented and discussed in Section S4 and Figure S4 in the Supplementary Materials.

**Figure 5:**
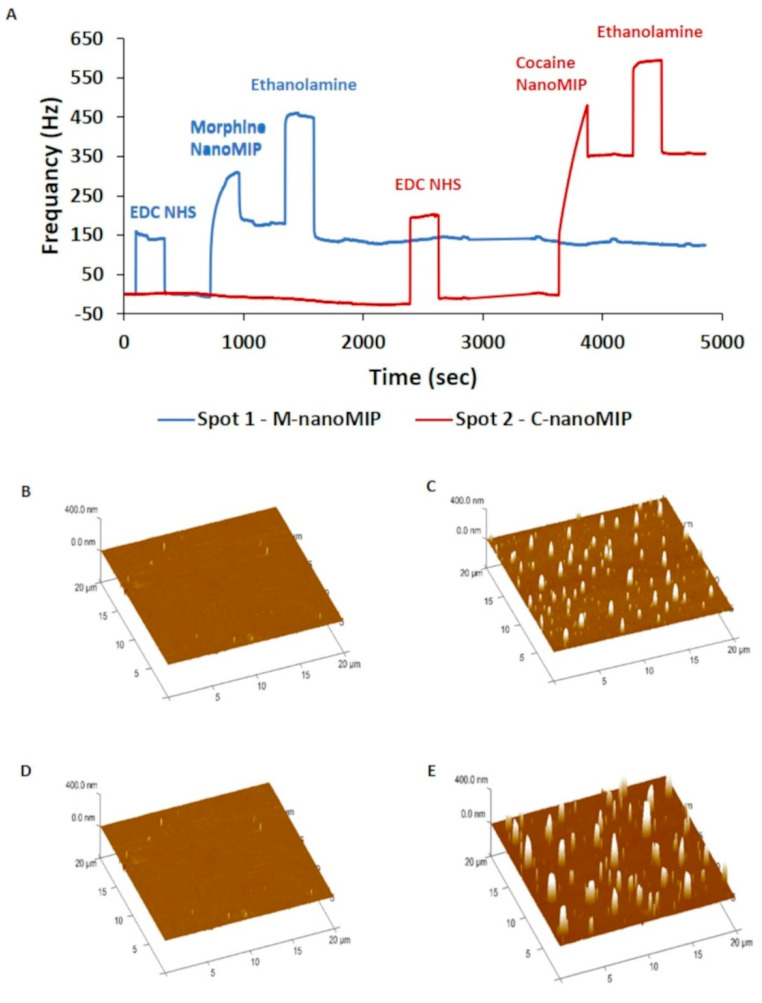
(**A**) Full sensorgram obtained during morphine and cocaine nanoMIP QCM sensor functionalization. AFM 3D topography image related to MUDA (**B**,**D**), morphine nanoMIP (**C**) and cocaine nanoMIP (**E**) functionalized spots of the gold QCM sensor surface (scan area = 400 µm^2^; height = 400 nm). White peak refers to the attached nanoMIP.

The gold surface of the QCMA-1 chip was cleaned using piranha solution to assure a homogenous modification of the sensor surface. Subsequently, a 11-MUDA monolayer was created onto the cleaned gold surface. Typically, this results in a layer of carbon chains with an inclination of 30° to the orthogonal line to the sensor chip surface [36]. Next, QCM sensor chip was docked in the QCM-1 instrument to perform the nanoMIP covalent attachment via amine coupling by EDC-NHS chemistry. This induced covalent attachment of the ammine functionalized nanoMIP onto the sensor chip (Figure 5). The concentration used was considered high enough to achieve the nanoMIP immobilization without affecting the instrument’s microfluidic system. A second injection was performed to assess whether the QCM spot surface was saturated entirely after the first injection of morphine nanoMIP. The result showed that the first injection saturated the surface (Section S5, Figure S5). Therefore, this concentration (1.2 mg·mL^−1^) was used for further investigation.

Similarly, the attachment of the cocaine nanoMIP on spot 2 of the QCM chip was investigated (Section S5, Figure S6). No further cocaine nanoMIP attachment was observed after the first injection at a concentration of 1.8 mg·mL^−1^. Following the attachment of both nanoMIP, ethanolamine was used to cap the unreacted groups and to prevent any non-specific binding events. The frequency responses for the morphine nanoMIP and cocaine nanoMIP attachments proved to be reproducible (5 replicates each) and equal to 251.81 ± 6.49 Hz and 358.60 ± 6.55 Hz, respectively. A typical sensorgram is displayed in Figure 5A. The difference in the frequency response between the morphine nanoMIP and the cocaine nanoMIP can be ascribed to the difference in the concentration used and the possible disparity in the molecular weight of the two nanoMIP.

AFM was used to confirm nanoMIP attachments onto the QCM chips surface. The 3D AFM images highlighted the difference in sensor surface before and after nanoMIP functionalization (Figure 5B–E). The microfluidic system allowed a uniform attachment of the nanoMIP on the QCM sensor surface, evidenced by the evenly distributed peaks. As per QCM surface roughness analysis performed by AFM, the average (nm SD±) Ra (3.10 ± 0.06) and Rq (1.88 ± 0.01) and Rmax (175.00 ± 32.53) values indicate that the MUDA was uniformly deposited onto the gold surface. The peaks detected on the QCM sensor spots refer to the nanoMIP, while the difference in peaks height and size can be due to the varying nanoMIP size or a possible nanoMIP aggregation. Overall, the roughness analysis confirmed the nanoMIP immobilization onto the QCM sensor surface. The results also showed that the Rq (37.30 nm), Ra (12.50 nm), and Rmax (503 nm) values of the cocaine nanoMIP functionalized surface was higher than the Rq (18.80 nm), Ra (6.01 nm), and Rmax (266 nm) values of the morphine nanoMIP functionalized surface. These results can be attributed to the size difference between the two types of nanoMIP. As displayed by the AFM images, the two spots were functionalized with a comparable amount of nanoMIP, despite the difference in the QCM frequency response. Similar results were achieved in the past when other types of sensing receptors were immobilized onto the QCM sensor chip [3].

#### 3.3.2. QCM Sensors Sensitivity and Specificity

**Figure 6:**
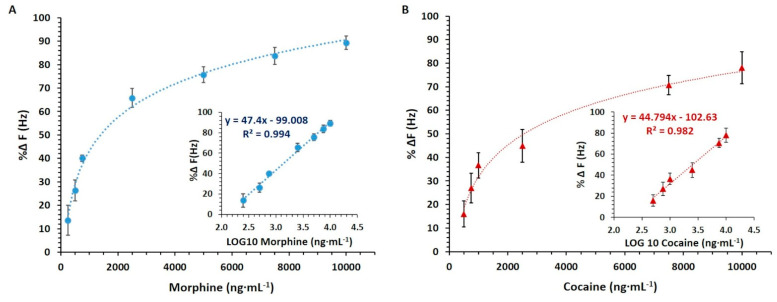
(**A**) Standardized Non-linear and linear calibration (inset) curves related to the morphine cumulative assay double-subtracted (blank and control signals subtracted). (**B**) Standardized non-linear and linear calibration (inset) curves related to the cocaine cumulative assay performed onto the nanoMIP QCM sensors. Error bars refer to the standard deviation of replicates (*n* = 3).

The sensitivity and specificity studies were performed using the multiplex nanoMIP QCM sensor. PBS was chosen over MOPS as running buffer due to the better signal-to-noise response. After priming the sensor surface, injections of 100 µL of the blank solution (blank AuNPs) on both spots 1 and 2 were performed until a stable blank signal was achieved. This provided the blank signal baseline and the evidence that no false response occurred due to the interaction between the blank AuNPs and the nanoMIP sensor surface. Then, morphine-AuNPs concentrations were injected from the lowest to the highest on spots 1 and 2. A typical sensorgram is presented in Section S5, Figure S7 in Supplementary Materials. The results showed that morphine nanoMIP sensor (spot 1) was proportionally responsive to the increasing concentration of the morphine AuNPs, while no apparent response was detectable on cocaine nanoMIP sensor (spot 2). A linear calibration curve was obtained (R^2^ = 0.994; *p*-value < 0.0001) by transforming the morphine concentrations into LOG_10_, as shown in Figure 6A. The LOD was found to be 0.19 µg·mL^−1^. The average (SD±) of the K_D_ was equal to 6.47 × 10^−8^ ± 3.40 × 10^−8^.

The nanoMIP QCM sensor’s ability to discriminate morphine from cocaine was investigated by performing the cocaine AuNPs cumulative assay. The sensor was responsive to the cocaine AuNPs on spot 2, whereas no response was observed on spot 1. A correlation (r = 0.998; *p*-value < 0.0001) between the ΔF (Hz) values and the increasing cocaine AuNPs concentrations was detected on spot 2. The averaged standardized sensors response (%F (Hz)) was plotted against the cocaine AuNPs concentrations, thus achieving the non-linear calibration curve (Figure 6A). The cocaine concentrations were then expressed as LOG_10_, and the linear calibration curve was obtained with an R^2^ equal to 0.978 (*p*-value < 0.0001) (Figure 6B). The LOD was calculated and was equal to 0.36 µg·mL^−1^. The average (SD±) of the K_D_ values were found to be 2.25 × 10^−7^ ± 1.9 × 10^−7^.

### 3.4. NanoMIP EIS and QCM Sensor Comparison

The work demonstrates that both sensor platforms were successfully functionalized with morphine nanoMIP and were able to detect morphine, a small molecule, at low concentration, with sensitivity and specificity. Furthermore, the work offers the opportunity to highlight the practical differences, pros and cons of EIS and QCM. Other than sensor working principle, significant differences were found in sensor platform design, portability, surface functionalization, assay design, data processing, analysis and platform sensitivity. Whereas some of the differences observed were linked to the two specific types of platforms used for the experiments, a few were general and related to the EIS and QCM techniques. 

The EIS equipment requires an electrode immersed into an electrolyte solution and connected to a potentiostat/galvanostat and a frequency response analyzer (FRA). The FRA/potentiostat system introduces the desired voltage between the WE and the electrolyte solution and concurrently records the current flowing between them. EIS FRA/potentiostat instrumentation used in this work (PalmSens 5, PalmSens BV) can be controlled remotely by a smartphone app (PStouch) through Bluetooth tethering and, therefore, can be considered a portable and flexible platform, amenable to “point of care” use. Furthermore, the instrument can be interfaced with a wide range of different electrodes (such as screen-printed and interdigitated electrodes). While the consecutive EIS reading is allowed, the simultaneous EIS measurements of two or more electrodes are an option not yet available within the PalmSens platform, thus slowing down the measurement’s workflow and limiting the development of a multiplex sensor. The electrodes handling and connection were also a bottleneck as great care was needed to avoid scratches or breakages and disconnection. A custom-made 3D printed CACIDE cable holder was designed to stabilize the connector-IDE interface. However, the brittleness of the glass substrate resulted in electrodes breakages. As such, we recommend the use of the more expensive ceramic-based IDE. Another drawback was the extensive time required to fit the raw data (expressed as imaginary, Z”, and real impedance, Z’) into the equivalent circuit needed to gather the actual sensor response measurement (Rct). By contrast, the QCM sensor platform used in this work (QCMA-1, Sierra Sensors GmbH, Hamburg, Germany) has a fully embedded microfluidic system and simultaneously works on two sensing spots. The QCM also allowed a time-solved visualization of the sensor response (*f*, Hz). Additionally, QCM raw data processing required minimal efforts as was carried out by the integrated software Sierra Sensor Analyser. However, the QCM machine selected for this work is primarily used to develop in-lab analytical methods and requires extensive time for the routine maintenance of the embedded microfluidic system.

Extensive EIS characterization studies were carried out on SPE and IDE surfaces at each fabrication step (bare, MUDA and nanoMIP and blocking agents’ functionalization). Overall, the EIS characterization results showed that the covalent attachment of morphine nanoMIP onto both types of gold electrodes (SPE and IDE) was achieved with good reproducibility. AFM analysis, performed in tapping mode, confirmed the success of the nanoMIP attachment and proved to be consistent with previous work related to the nanoMIP or antibodies attachment onto gold sensor surfaces [37,38]. However, the roughness analysis was less informative due to the IDE’s geometrical conformation and the irregularities on the WE of the SPE, which introduced a bias on the values of the roughness surface parameters (Ra, Rq and Rmax). Analogously, the nanoMIP QCM sensor fabrication was visualized by QCM and investigated further by AFM. The success of the surface functionalization was readily visible on the sensorgram and proved to be reproducible. Furthermore, AFM images and roughness analysis showed a homogeneous nanoMIP deposition onto the gold surface and were comparable to AFM images of other nanoMIP attachments onto similar gold sensors surfaces [38]. The smoother and regular gold surface of QCM chips and the use of the microfluidic system resulted in faster (few minutes) and more uniform surface functionalization compared to the longer (several hours) and heterogeneous nanoMIP surface coverage observed on IDE and SPE electrodes.

The EIS sensor outperformed the QCM sensor in terms of sensitivity and assay design in this work. The direct and label-free assay was able to detect a small molecule, namely morphine, at trace levels. Notably, the LOD of morphine nanoMIP EIS sensor developed onto SPE (0.11 ng·mL^−1^) was equal to the LOD achieved when the same sensor was fabricated on IDE (0.11 ng·mL^−1^). Although reported in the literature [25], the results of this work do not provide evidence of higher sensitivity in using IDE electrodes. However, the linear ranges of nanoMIP sensor fabricated on IDE occurred at lower concentrations than the linear ranges of the sensors fabricated on SPE. Thus, it is likely that the IDE sensor’s sensitivity may improve by performing further optimization studies, such as using a different nanoMIP concentration or EIS settings.

Overall, the results indicated that the morphine nanoMIP EIS sensor (using SPE) can specifically detect morphine without cross-reacting with cocaine and paracetamol. Compared to EIS, the QCM biosensor platform is not as sensitive for detecting small molecules, as no signal was recorded when concentrations of morphine in the ng-µg·mL^−1^ were used for the binding assay. Consequently, the analyte’s conjugation to the gold nanoparticles (AuNPs) was required to increase the mass of morphine and enhance the QCM sensor sensitivity. Although the assay design can be described as direct, the development of a competitive assay or a sample purification and conjugation protocol is required for the application with real-samples. Overall, drugs were adsorbed onto the gold nanoparticles and were detected with a LOD equal to 0.19 µg·mL^−1^. The work also demonstrated that the nanoMIP QCM sensor could differentiate cocaine from morphine and is suitable for a multiplex sensor platform. The opportunity to follow the adsorption and desorption phenomena allowed us to determine the affinity (K_D_) between the morphine nanoMIP and the morphine and cocaine conjugated to AuNPs, which were equal to 6.47 × 10^−8^ ± 3.40 × 10^−8^ and 2.25 × 10^−7^ ± 1.9 × 10^−7^, respectively.

The higher sensitivity of EIS biosensors is counterbalanced by the occurrence of non-specific binding in the EIS sensor surface. Therefore, the EIS sensor requires an additional blocking agent (i.e., 0.1% BSA–1% Tween 20) compared to the QCM sensor surface. This might be attributed to a longer incubation time (30 min) and manual washing of the EIS sensor compared to the shorter injection time (4 min), the consistency of the washing step on the QCM sensor surface use of the microfluidic system. To conclude, it is worth mentioning the recent development of an innovative electrochemical–quartz crystal microbalance (IEQCM) sensor [39], which promises to exploit each platform’s benefits and overcome their drawbacks.

## 4. Conclusions

This work has compared the performance of two affinity sensing platforms, Faradic EIS and QCM, having nanoMIP as an artificial receptor and morphine as a target analyte. NanoMIP EIS sensor demonstrated outstanding performance in specifically detecting morphine at a low concentration in a direct assay format. Notably, the LOD of morphine nanoMIP EIS sensor developed onto SPE (0.11 ng·mL^−1^) was equal to the LOD achieved when the same sensor was fabricated using IDE (0.11 ng·mL^−1^). An analogous nanoMIP functionalization was applied onto QCM sensor chips and evaluated using a QCM instrument with a fully embedded microfluidic system. Prior to the binding, the drug was adsorbed onto gold nanoparticles to enhance the QCM sensitivity and to obtain a LOD equal to 0.19 µg·mL^−1^. However, EIS sensors detected morphine at a concentration lower than three orders of magnitude compared to QCM sensor. Overall, both QCM and EIS specifically detected morphine in a direct assay format, although QCM required morphine AuNPs conjugation to obtain a successful detection on the µg·mL^−1^ range. On the other hand, EIS required extensive experimental time and data processing, resulting in longer analysis lapse time. In addition, presently, EIS does not allow to observe the receptor-analyte binding in a real-time, as measurements are carried out post-binding (at least when a redox probe is used for the readings). Contrariwise, QCM permitted the recording of real-time binding data and the estimation of the equilibrium binding constant K_D_. The work also highlighted the advantage of using our QCM instrument as a multiplex sensing platform compared to EIS. 

To conclude, the EIS sensor platform is portable and can achieve an outstanding sensitivity. As such, it might be better suited for in-field operation. On the other hand, the QCM sensor used in this work, is a bulky piece of equipment and more indicated for in-lab and multiplex detection methods as it requires sample preparation and proper equipment maintenance. Nevertheless, this work has shown that both platforms can detect illicit drugs, such as morphine, and particularly the EIS nanoMIP sensor could be developed further and validated with real samples.

## Figures and Tables

**Table 1:** Summary of the statistical results of equivalent circuit fittings and calibration curves of nanoMIP EIS-SPE Sensors.

pH	Buffer	Analyte	Equivalent Circuit Fitting				Calibration Curve			
			Average Rct Error (%)	SD ± (%)	χ^2^	SD±	*n*	r	R^2^	*p*-Value
6.0	MOPS	Morphine	6.01	1.83	0.008	0.001	9	0.429	0.184	0.250
7.4	MOPS	Morphine	2.94	0.58	0.012	0.005	7	0.986	0.977	<0.0001
9.0	PBS	Morphine	5.12	2.17	0.002	0.001	8	0.950	0.020	0.823
7.4	MOPS	Morphine–no nanoMIP	2.47	0.83	0.008	0.005	5	0.600	0.404	0.250
7.4	MOPS	Cocaine	1.83	0.45	0.018	0.007	5	0.053	0.003	0.892
7.4	MOPS	Paracetamol	3.22	0.81	0.014	0.006	8	0.215	0.046	0.911

SD = standard deviation; χ^2^ = chi squared; *n* = number of independent replicates; r = correlation coefficient.

## Data Availability

Data available on request due to restrictions e.g. privacy or ethical.

## Supplementary Materials

The following are available online at https://www.mdpi.com/article/10.3390/nano11123360/s1, Figure S1. (A) TEM image of morphine nanoMIP batch 2. (B) Schematic diameter measurements recorded for each nanoMIP captured by TEM analysis. (C) Bar chart of each measured nanoMIP diameter. Error bars refer to the SD± of measurement recorded along different nanoMIP axes, Figure S2. D model of the DPR IDEAu5 connector: (A) holder and (B) cover realized using SketchUp® software, Figure S3. (A) Final 3D printed DPR IDEAu5 holder (front view). (B1) Final 3D printed DPR IDEAu5 holder and (B2) holder cover (top view). (B3) 3D printed DPR IDEAu5 holder entirely enclosed by the holder cover. (C) 3D printed DPR IDEAu5 holder placed inside the Faraday cage, Figure S4. (a): (A) Bare gold nanoparticle; (B) Blank gold nanoparticle (Blank AuNP), blocked with BSA (Bovine Serum Albumin); (C) Cocaine conjugated gold nanoparticles (cocaine AuNP); (D) Morphine conjugate gold nanoparticle (morphine-AuNP); (b): Average of *d*_H_ values observed for bare AuNP, Blank AuNP and morphine conjugated AuNP (MO AuNP) and cocaine conjugated AuNP (CO AuNP) obtained by DLS analysis. Error bars refer to the standard of replicates (n = 10). The inset table reports the average (SD±) of the *d*_H_ and corresponding PDI values, Figure S5. Sensorgram of morphine nanoMIP attachment onto spot 1 performed by two consecutive nanoMIP injections at a concentration of 1.2 mg·mL^−1^. No significant attachment was achieved after the first injection, Figure S6. Sensorgram of cocaine nanoMIP attachment onto spot 1 performed by two consecutive nanoMIP injections at a concentration of 1.8 mg·mL^−1^; no significant attachment was achieved after the first injection, Figure S7. Sensorgram obtained during the morphine cumulative assay. The nanoMIPs QCM was operating in morphine sensing mode. The increase sensor response to morphine AuNPs is visible on spot 1 (= active spot), Table S1. (A) Average value (SD±) of hydrodynamic diameter (*d*_H_) and polydispersity index (PDI) across the different morphine nanoMIP batches obtained during DLS analysis. (B) Results of the One-way ANOVA and Post Hoc Scheffé’s test obtained by comparing the *d*_H_ of each morphine nanoMIP batch.
