# Supplementary material for: A Comparison of EIS and QCM NanoMIP-Based Sensors for Morphine
Source: Nanomaterials (Basel). 2021 Dec 11;11(12):3360. doi: 10.3390/nano11123360 (PMC8707575; doi:10.3390/nano11123360)
Supplement: Supplementary file 1 [file nanomaterials-11-03360-s001.zip › nanomaterials-1491480-supplementary-revised.pdf]

nanomaterials

Supplementary Materials

A Comparison of EIS and QCM NanoMIP-Based Sensors for Morphine

Roberta D'Aurelio [1,*], Ibtisam E. Tothill [1], Maria Salbini [1,2], Francesca Calò [1,2], Elisabetta Mazzotta [2], Cosimino Malitesta [2] and Iva Chianella [1,*]

[1] Surface Engineering and Precision Centre, School of Aerospace, Transport and Manufacturing, Cranfield University, Cranfield, Bedfordshire MK43 0AL, England, UK; i.tothill@cranfield.ac.uk (I.E.T.); maria.salbini@nanotec.cnr.it (M.S.); francesca.calo@unisalento.it (F.C.)

[2] Laboratorio di Chimica Analitica, Edificio Multipiano CSEEM A6., Dipartimento di Scienze e Tecnologie Biologiche ed Ambientali, Università del Salento, I-73100 Lecce, Italy; elisabetta.mazzotta@unisalento.it (E.M.); cosimino.malitesta@unisalento.it (C.M.)

* Correspondence: r.daurelio@cranfield.ac.uk (R.D.); i.chianella.1998@cranfield.ac.uk (I.C.)

S1. Apparatus and Measurements

NanoMIP Characterization: NanoMIPs and gold nanoparticles used in this work were characterized using Transmission Electron Microscopy (TEM, Phillips, CM20, Philips Electronics, Eindhoven, The Netherlands) and Dynamic Light Scattering (DLS - Zetasizer Nano-S, Malvern Panalytical Ltd, Malvern, UK). To perform the DLS analysis, the nanoMIP solvent (acetonitrile) was exchanged to water via Eppendorf concentrator 5301 (Eppendorf UK Ltd, Stevenage UK), and was then filtered using a 0.45 µm diameter filter. In order to perform the TEM analysis, 10 µL of nanoMIPs solution was deposited on the TEM sample copper holder and the solvent was allowed to evaporate. The Full sample preparation description is reported in Section S2).

EIS: PalmSens4 (PalmSens BV, Houten, The Netherlands) was purchased from Alvatek Ltd (Tetbury, UK) and used as potentiostat/ Electrochemical Impedance Spectroscopy (EIS) analyser. The PalmSens was connected to a PC on which the dedicated PStrace 5 version 5.8.1704 (Software for data acquisition and processing, PalmSens BV, Houten, The Netherlands) was installed. Two types of commercially available electrodes were used in this work:

i. DropeSens screen-printed electrodes (SPEs), DPR – C220AT (DropSens S.L., Llanera, Spain) purchased from Metrohm (Runcorn, UK);
ii. DropSense interdigitated electrodes (IDEs), DRP-G-IDEAU5 (DropSens S.L., Llanera, Spain) also purchased from Metrohm (Runcorn, UK).

The DropSens SPE. was connected *via* an universal sensor connector (PalmSens®, supplied by Alvatek Ltd, Tetbury UK), which was coupled to the instrument by a double-shielded cable with a 2 mm banana connectors (PalmSens®, supplied by Alvatek Ltd, Tetbury UK). DropSens IDE was connected to the instrument by a double-shielded cable CACIDE (DropSens S.L., Llanera, Spain), courtesy modified by PalmSens BV. An IDE custom-made connector holder was designed and 3D printed to allocate the CACIDE connectors. Specifically, the holder and the cover were designed using the 3D modelling computer program (SketchUp®, Trimble Inc., Sunnyvale, CA, USA). The holder was then manufactured at the Department Centre for Engineering Photonics using a 3D printer (Ultimaker 2+, Ultimaker B.V, Geldermalsen, The Netherlands). The holder was printed with a P.L.A. Innofil 3D 2.85 mm (Innofil 3D BV, Emmen, The Netherlands). The connectors were placed into a VistaShield™ Faraday Cage (Gamry Instruments, Warminstercity, PA, USA) during the recording of all EIS measurements to reduce the environmental, electrical noise. Stainless steel and plastic tweezers were used to handle all the electrodes.

The EIS measurements were performed in a frequency range from 50 kHz to 0.1 Hz, with a Direct Current (DC) equal to 0.12 V and a stimulus wave of 10 mV. All EIS measurements were performed in 10 mM redox couple solution ($[Fe(CN)_6]^{3-/4-}$) and at room temperature (23 °C ± 1). The redox couple solution was composed by potassium hexacyanoferrate III (K3$[Fe(CN)_6]$) and potassium hexacyanoferrate(II)trihydrate ($K_4[Fe(CN)_6]3H_2O$), dissolved in MOPS (10 mM, pH 7.4). A 50 µL of 10 mM redox couple solution ($[Fe(CN)_6]^{3-/4-}$) (pH = 7.4) was dropped onto the Dropsens SPE. before starting the EIS measurement. The volume of the redox solution was increased to 60 µL for the DropsSens IDE. The experimental data were fitted onto an appropriate equivalent circuit by EIS spectrum analyser® v1.0 (Software for data processing, Bondarenko A. S., Ragoisha G. A. In Progress in Chemometrics Research, Pomerantsev A. L., Ed.; Nova Science Publishers: New York, 2005). Briefly, the Faradaic EIS spectrum of a sensor surface appears as a semicircle followed (or not) by a straight line in the Nyquist plot and, therefore is described by Randles or simplified Randles equivalent circuit, respectively.

QCM: QCMA-1 affinity sensors chip and the biosensor instrument (QCMA1) with embedded automatic microfluidic system used in this work were purchased from Sierra Sensors GmbH (Hamburg, Germany). The dedicated software Sierra Analyser v.3.1.10.0 (Software for data processing, Sierra Sensors GmbH, Germany) was used to acquire and process the experimental data.

Sensors Surface Characterization: Atomic Force Microscopy (AFM, Dimension 3100, Bruker, Coventry, UK) was used to characterize both the EIS and QCM sensor surfaces. The AFM analysis was carried out in tapping mode. Prior to the analysis, the sensors surfaces were thoroughly rinsed with deionized water, filtered through a 0.2 µm syringe filter, and dried under a gentle stream of nitrogen. Following each functionalization step, the SPEs and IDEs sensors surface were characterized by recording EIS measurements. The obtained EIS data were fitted in the appropriate equivalent circuit. The Rct was expressed as a percentage of the MUDA coated electrodes' average Rct value (set as a 100%).

S2. NanoMIPs Characterization

DLS analysis: DLS analysis was carried out using Zetasizer Nano (Nano-S, Malvern Panalytical Ltd, Malvern, UK). A 1 mL of diluted nanoMIP in water samples were analyzed in 3 cm^3 disposable polystyrene cuvettes at 25 °C, having polystyrene latex as the reference material. At least 10 measurements were recorded for each batch (Table S1).

Table S1. (**A**) Average value (SD±) of hydrodynamic diameter (d_H) and polydispersity index (PDI) across the different morphine nanoMIP batches obtained during DLS analysis. (**B**) Results of the One-way ANOVA and Post Hoc Scheffé's test obtained by comparing the d_H of each morphine nanoMIP batch.

A

	**Morphine nanoMIP**			
	d_H		**PDI**	
**Batch**	**Average**	**SD±**	**Average**	**SD±**
1	130.59	3.12	0.13	0.014
2	268.39	37.39	0.23	0.03
3	163.45	2.66	0.09	0.02
4	124.57	5.44	0.27	0.01

B

			**Batch/d_H(nm)**		
			**Subset for alpha = 0.05**		
	**Batch**	**N**	**1**	**2**	**3**
	1	10	130.59		
	4	10	124.57		
Scheffe[a,b]	3	20		163.44	
	2	10			268.39
	Sig.		0.866	1.000	1.000

Means for groups in homogeneous subsets are displayed.
a. Uses Harmonic Mean Sample Size = 11.429.
b. The group sizes are unequal. The harmonic mean of the group sizes is used. Type I error levels are not guaranteed.

TEM analysis. The nanoMIP samples were prepared by filtering 1 mL of the nanoMIPs solution using a 0.45 syringe filter. Then, 10 μL of the nanoMIP solution was placed on a silicon chip attached to a TEM holder and left to dry overnight in a fume hood. Software-assisted image processing approach was used to evaluate the diameter size of both the cocaine and morphine nanoMIP. Specifically, the TEM images were analyzed by ImageJ® version 1.52u (software for image analysis, National Institutes of Health, Bethesda, MD, USA, 2020). Briefly, measurements were taken along the four-axis of the nanoparticle's circumference for each numbered nanoMIP (i.e., vertical, horizontal and oblique diameters), as illustrated in Figure S1. The measurements were then averaged and expressed as the average diameter size (SD±). Batch 2 was used as an example in Figure S1.

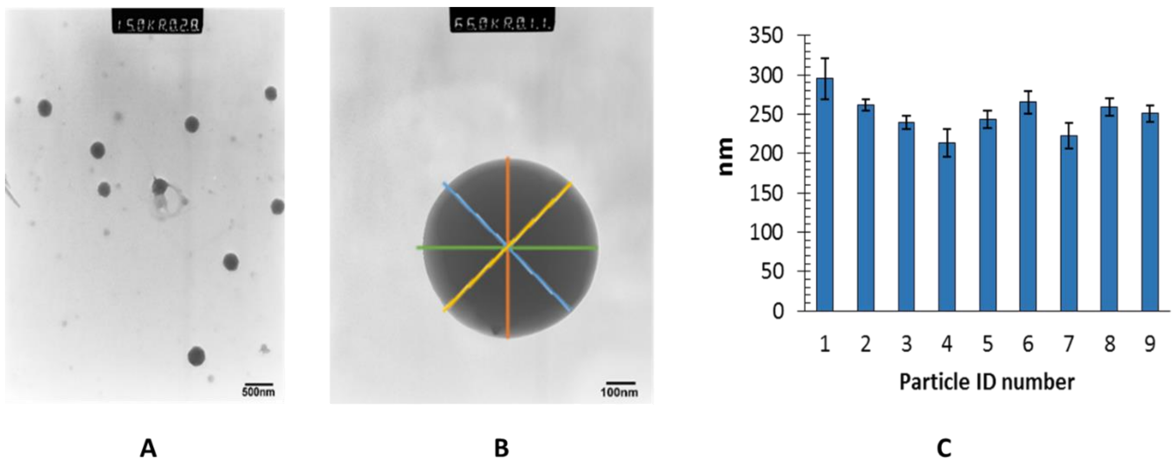

Figure S1. (**A**) TEM image of morphine nanoMIP batch 2. (**B**) Schematic diameter measurements recorded for each nanoMIP captured by TEM analysis. (**C**) Bar chart of each measured nanoMIP diameter. Error bars refer to the SD± of measurement recorded along different nanoMIP axes.

S3. Design of the 3D Printed And Custom-Made Connector

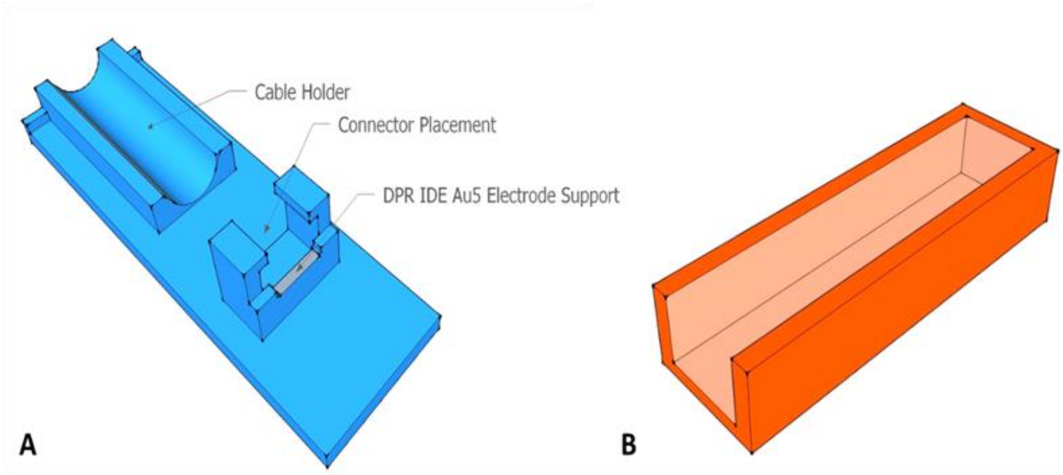

Figure S2. D model of the DPR IDEAu5 connector: (**A**) holder and (**B**) cover realized using SketchUp® software.

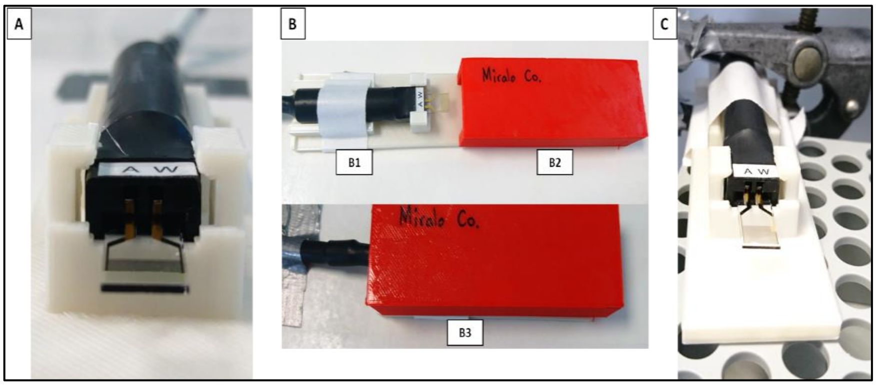

Figure S3. (**A**) Final 3D printed DPR IDEAu5 holder (front view). (**B1**) Final 3D printed DPR IDEAu5 holder and (**B2**) holder cover (top view). (**B3**) 3D printed DPR IDEAu5 holder entirely enclosed by the holder cover. (**C**) 3D printed DPR IDEAu5 holder placed inside the Faraday cage.

S4. Drugs– AuNP conjugation

Briefly, a minimum of ten DLS measurements of the bare AuNPs, the blank, the cocaine and morphine conjugated AuNPs were recorded. The one-way ANOVA test with Sheffe's post hoc test revealed d_H differences among the samples, as shown in Figure S4.

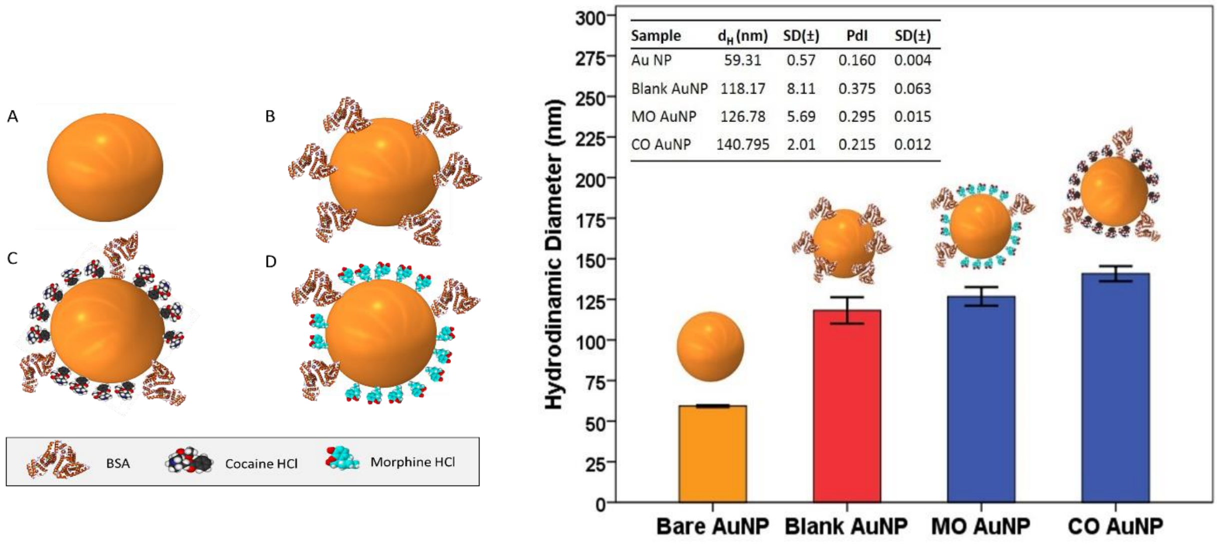

(**a**) (**b**)

Figure S4. Figure (**a**): (**A**) Bare gold nanoparticle; (**B**) Blank gold nanoparticle (Blank AuNP), blocked with BSA (Bovine Serum Albumin); (**C**) Cocaine conjugated gold nanoparticles (cocaine AuNP); (**D**) Morphine conjugate gold nanoparticle (morphine-AuNP); Figure (**b**): Average of d_H values observed for bare AuNP, Blank AuNP and morphine conjugated AuNP (MO AuNP) and cocaine conjugated AuNP (CO AuNP) obtained by DLS analysis. Error bars refer to the standard of replicates (n = 10). The inset table reports the average (SD±) of the d_H and corresponding PDI values.

Specifically, the d_H of the blank AuNPs (118.17 ± 8.11 nm) was bigger than the bare AuNPs (59.31 ± 0.57 nm) and slightly smaller than the morphine conjugated AuNPs (126.78 ± 5.69 nm). Interestingly, the bare AuNPs average d_H size was slightly bigger than the 40 nm declared by the manufacturer, and this is possible due to spontaneous AuNPs aggregation occurring in water solution. The one-way ANOVA test confirmed that the differences between the bare, blank, and morphine conjugated AuNP samples were significant (F (2,38) = 535.071; *p*-value < 0.00001). Furthermore, the Sheffe's post hoc test revealed that, on average, morphine AuNPs were 67.47 nm and 8.60 nm bigger than the AuNPs and the blank AuNPs, respectively. Similarly, the average (SD±) values of the d_H of the bare, blank and cocaine conjugated AuNP (140.80 ± 2.51 nm) were statistically different according to the one-way ANOVA (F (2,47) = 1731.997, *p*-value < 0.00001). The Sheffle's post hoc test revealed that the d_H (nm) size of cocaine AuNPs was 22.26 nm bigger than the blank AuNPs (*p*-value < 0.00001) and 81.46 nm bigger than the bare AuNPs (*p*-value < 0.00001).

S5. QCM sensorgrams

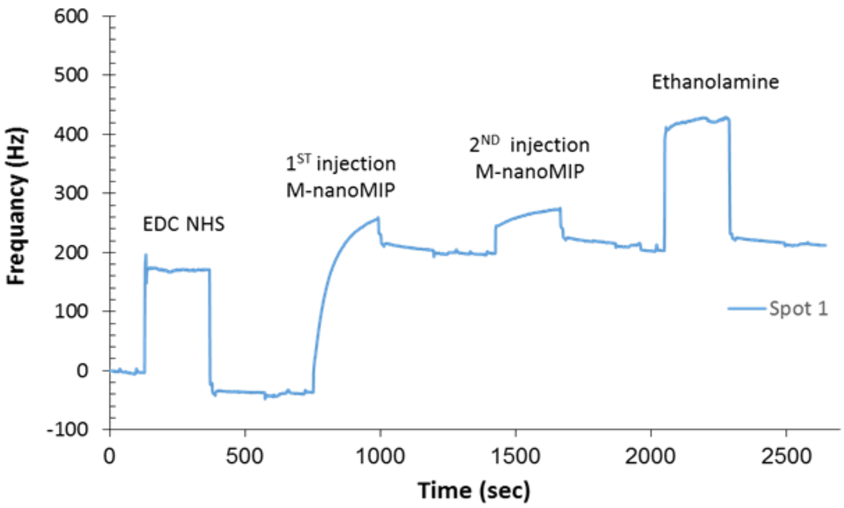

Figure S5. Sensorgram of morphine nanoMIP attachment onto spot 1 performed by two consecutive nanoMIP injections at a concentration of 1.2 mg·mL^{-1}. No significant attachment was achieved after the first injection.

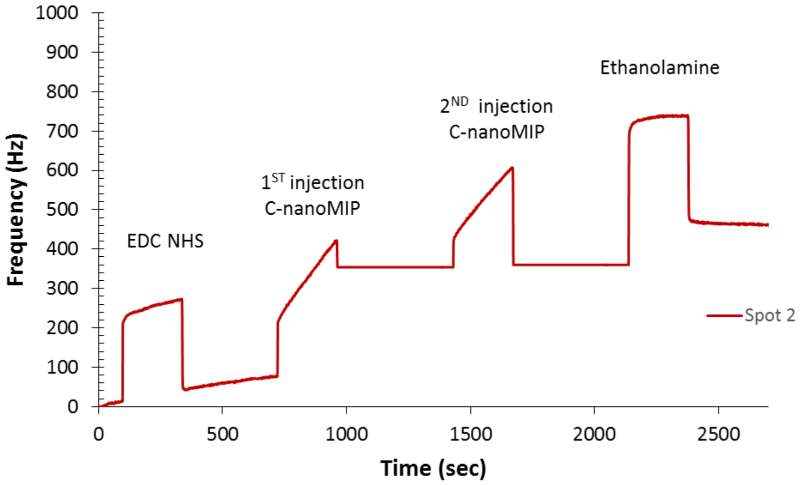

Figure S6. Sensorgram of cocaine nanoMIP attachment onto spot 1 performed by two consecutive nanoMIP injections at a concentration of 1.8 mg·mL^{-1}; no significant attachment was achieved after the first injection.

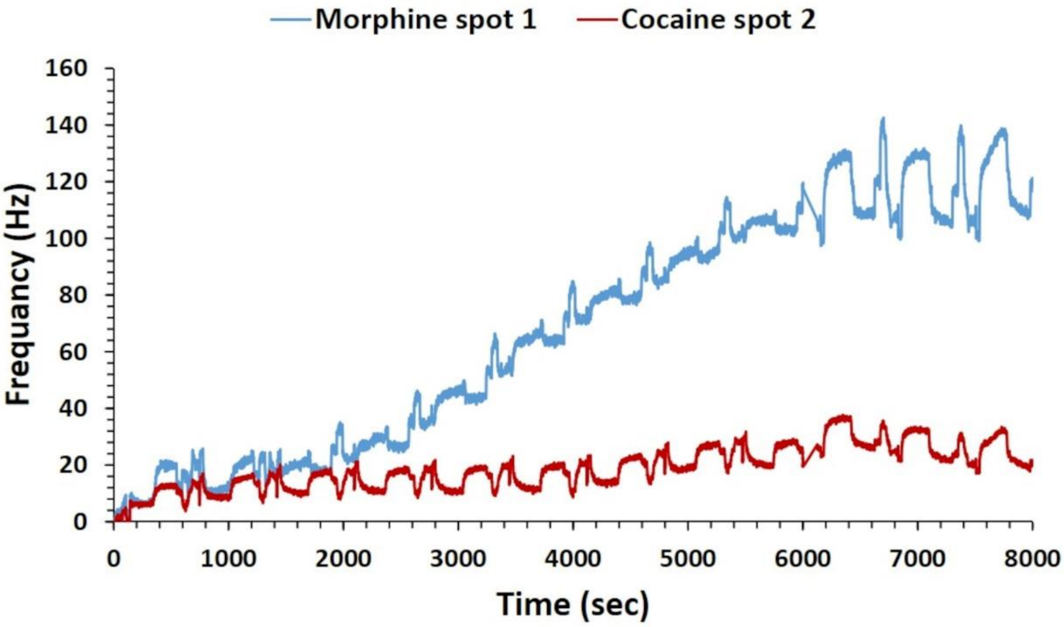

142

Figure S7. Sensorgram obtained during the morphine cumulative assay. The nanoMIPs QCM was operating in morphine 143
sensing mode. The increase sensor response to morphine AuNPs is visible on spot 1 (= active spot). 144
